# A novel real-time intravascular ultrasound double-lumen microcatheter for recanalization of chronic total occlusion: a case report

**DOI:** 10.1186/s13256-019-2230-5

**Published:** 2019-10-23

**Authors:** Yanzhuo Ma, Yuhong Peng, Gang Wang, Leisheng Ru

**Affiliations:** Department of Cardiology, Bethune International Peace Hospital, Shijiazhuang, Hebei China

**Keywords:** Percutaneous coronary intervention, Chronic total occlusion, Catheter design, Intravascular ultrasound

## Abstract

**Background:**

Chronic total occlusion revascularization remains a challenging problem because of its complexity. We present a case of a patient with chronic total occlusion who was successfully revascularized with the use of a new device called a real-time intravascular ultrasound double-lumen microcatheter.

**Case presentation:**

A 58-year-old East Asians woman presented to our hospital with a complaint of recurrent chest pain of 5 months’ duration. Angiography revealed chronic total occlusion of the right coronary artery from the right coronary artery ostium to the ostia of the posterolateral and posterior descending branches. A guidewire was passed to the distal right coronary artery but went into the false lumens at the posterior descending and posterolateral ostia after use of the antegrade and retrograde approaches. Hence, we used the new device to pass through the subintimal right coronary artery space with reentry into the true lumen before the posterior descending and posterolateral ostia. A stent was successfully deployed at the posterior descending and posterolateral ostia, and the final result was excellent.

**Conclusions:**

This device was useful for finding the entry point and for reentry into the true lumen of a chronic total occlusion. It may be a valuable tool for recanalization of complex chronic total occlusion lesions.

## Background

The revascularization procedure for chronic total occlusion (CTO) is difficult and has a high risk of failure, so investigators have continued to seek to identify the optimal management strategy. We have been using a specially designed device consisting of an intravascular ultrasound (IVUS) catheter and a microcatheter for the crossing guidewire, which we call a real-time IVUS double-lumen microcatheter. This device has made it easier to find the entry point and to reenter the true lumen in CTO procedures. We present a case report to illustrate the usefulness of this catheter and to describe how it works and when to use it in CTO procedures.

### Catheter system

**Fig. 1:**
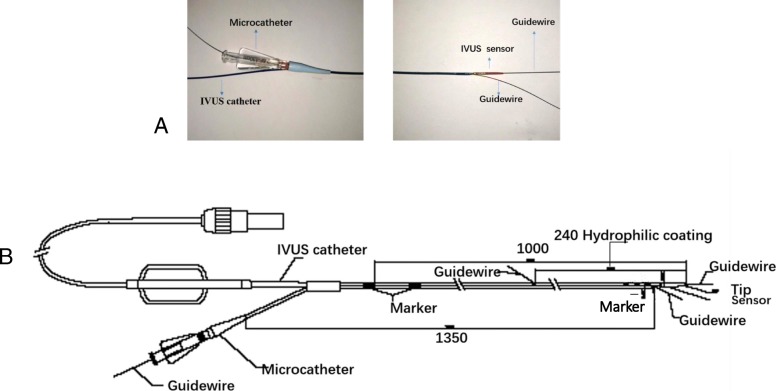
Catheter system. **a** Photograph of the device; **b** Design drawing of the device

The real-time IVUS double-lumen microcatheter consists of an IVUS catheter and a microcatheter. The device was designed by us (patent number ZL 2014 20,322,858.5) and manufactured by APT Medical (Hunan, China). The IVUS catheter serves as a monorail lumen, helps to support and stabilize the guidewire in the microcatheter, and provides real-time images. The microcatheter serves as an over-the-wire (OTW) lumen to pass a guidewire across the CTO lesion through the side hole (Fig. 1a, b). The length of this device is 1350 mm; the side hole of the OTW lumen is positioned 20 mm proximal to the distal tip; and the exit port of the monorail lumen is situated 240 mm proximal to the distal tip of the IVUS catheter. Three radiopaque markers with 10-mm spacing are situated to identify the exit ports of both lumens. Radiopaque markers are positioned 1 mm proximal to the side hole of the OTW lumen. The frequency of the IVUS in this device is 20 MHz, and there is a 240-mm-long hydrophilic coating that promotes smooth delivery. The widest diameter at the level of the two lumens is 3.6 French, and the outer diameter of this device mandates that the device can be used in a 6-French or larger guiding catheter. In clinical practice, when the first guidewire is crossed to the side branch or subintimal space and the device is advanced to the side branch or subintimal space via the first guidewire, the second guidewire is then passed through the side hole, crossing the entry point or entering into the true lumen according to the images provided by IVUS.

## Case presentation

**Fig. 2:**
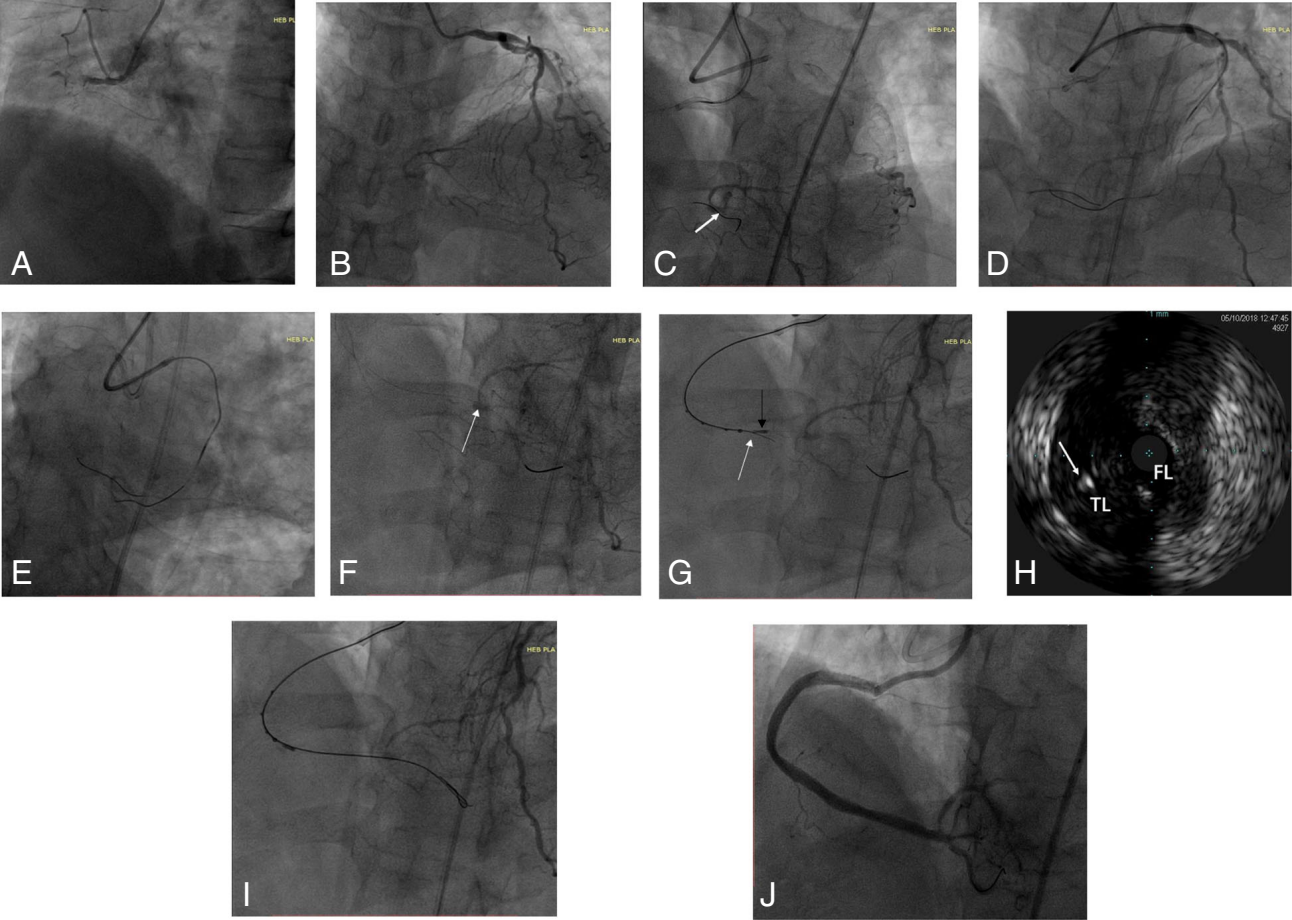
Angiographic and intravascular ultrasound (IVUS) images. **a** and **b** Middle and distal left anterior descending arteries with 50–60% stenosis, distal Circumflex artery (CX) with 60% stenosis, and chronic total occlusion of the right coronary artery (RCA) from RCA ostium to the ostia of the posterolateral (PL) and posterior descending (PD) branches. **c** The guidewire is in the subintimal space (white arrow). **d** The SION™ wire is passed to the septal branch via a Corsair™ 150 tube, but the SION™ wire and the antegrade wire are not touching. **e** The SION™ wire is passed to the epicardial branch via a Corsair™ 150 tube, but the SION™ wire and the antegrade wire are not touching. **f** The Gaia Third™ wire appears to have passed the PD ostium under the plaque (white arrow) after use of the parallel-wire technique. **g** The real-time IVUS double-lumen microcatheter is advanced via a Gaia Third™ wire to find the true lumen before the ostia of the PD and PL branches, and the Conquest Pro™ wire is punched into the true lumen before the ostia of the PD and PL branches under the guidance of real-time IVUS. White arrow, Conquest Pro wire; black arrow, IVUS probe. **h** IVUS image showing the Conquest Pro™ wire punctured into the true lumen under the guidance of real time IVUS (white arrow). **i** The Conquest Pro™ wire is passed to the distal PD branch. **j** The final angiographic result was excellent. *FL* False lumen, *TL* Total lumen

A 58-year-old East Asians woman with a history of diabetes was referred to our hospital because of recurrent chest pain of 5 months’ duration. She had undergone coronary computed tomographic angiography in our hospital 1 week earlier, which showed severe stenosis and calcification of the right coronary artery (RCA). Angiography revealed 50–60% stenosis in the middle and distal left anterior descending (LAD) arteries, 60% stenosis in the distal left circumflex artery, and CTO of the RCA from the ostium to the posterolateral (PL) branch and posterior descending (PD) branch ostia (Fig. 2a, b). Biradial percutaneous coronary intervention (PCI) in the RCA was performed by using a 7F AL1™ guide catheter for the RCA (Cordis, Santa Clara, CA, USA) and a 6-French EBU3.5™ guide catheter for the LAD (Medtronic Inc., Minneapolis, MN, USA). We intended to use the antegrade approach first and were able to pass a Fielder XT-R™ wire (Asahi Intecc Co. Ltd., Aichi, Japan) together with a Corsair™ 150 tube (Asahi Intecc Co. Ltd.) through the proximal RCA without difficulty, but it was hard to manipulate the guidewire in the proper direction. Next, a Gaia Second™ wire Miracle 6™ wire and a Conquest Pro™ wire (Asahi Intecc Co. Ltd.) were used to cross the occlusion lesion; however, the guidewire was shown to be in the subintimal space (Fig. 2c). Therefore, we aborted the antegrade approach and attempted the retrograde approach. A SION™ wire (Asahi Intecc Co. Ltd.) was passed to the septal branch via a Corsair™ 150 tube first, but it went into the false lumen no matter how many times we tried (Fig. 2d). We used the epicardial branch next but still failed (Fig. 2e). In this situation, we attempted the antegrade approach again, so a Crusade™ tube (Terumo, Tokyo, Japan) was introduced to the RCA via the previously placed Conquest Pro™ wire, and then a Gaia Third™ wire (Asahi Intecc Co. Ltd.) was passed via the side hole of the Crusade™ tube to the distal PD branch by using the parallel-wire technique. The Crusade™ tube was withdrawn, and the Corsair™ 150 tube was advanced. Blood was observed when we aspirated back through the syringe connected to the end of the Corsair™ tube, which indicated that the guidewire was in the true lumen of the distal side of the PD branch. Nevertheless, the Gaia Third™ wire appeared to have passed the PD branch ostium under the plaque (Fig. 2f), so the PL branch would have been unavailable if we had deployed the stent. Hence, we advanced our special device, the real-time IVUS double-lumen microcatheter, via the Gaia Third™ wire previously placed in the false lumen to find the true lumen before the ostia of the PD and PL branches. Figure 2g and h shows the IVUS probe being introduced into the false lumen over the Gaia Third™ wire. The device was then pulled back to find the true lumen. The Conquest Pro™ wire was then advanced through the microcatheter to push into the true lumen before the ostia of the PD and PL branches under the guidance of real-time IVUS, and the Conquest Pro™ wire was passed to the distal PD branch (Fig. 2i). Then, the IVUS was inserted into the RCA, and the IVUS showed that the guidewire was in the true lumen of the ostia of the PD and PL branches and was mostly in the subintimal space of the middle to distal RCA. Next, we performed balloon dilation with a 3.0 × 15-mm Maverick™ balloon (Boston Scientific, Marlborough, MA, USA) and deployed three 3.5 × 36-mm stents and one 4.5 × 14-mm Excel® stent (JW Medical Systems, Weihai, Shandong, China) without difficulty. The final angiographic result was excellent (Fig. 2j).

This study was approved by the Ethics Committee of the Bethune International Peace Hospital and was conducted according to the ethical principles of the Declaration of Helsinki. All of the enrolled patients provided written informed consent before the real-time IVUS double-lumen microcatheter was used in their treatment.

## Discussion and conclusion

It has been reported that CTO recanalization helps improve cardiac function and long-term survival [1–3], but CTO revascularization is a challenging technique [4, 5]. The inability of the guidewire to cross into the true lumen is the most common reason for failed CTO PCI. Improvements in devices and technology have significantly increased successful CTO recanalization rates [6–8]. The use of double-lumen microcatheters and IVUS has made it easier to find the entry point and the true lumen, thus increasing the rate of successful recanalization and decreasing the rate of complications during these procedures [9].

A double-lumen microcatheter has a type of construction that is effective for providing support and pushing. The guidewire in the monorail lumen protruding from the end hole helps to stabilize the device, and the guidewire in the OTW lumen protruding from the side hole can be directed toward the intended lesion. In other words, this device provides adjunctive support to facilitate placement of the guidewire in the desired position and prevent entanglement of multiple guidewires [10, 11].

It has been demonstrated that IVUS-guided CTO intervention improved clinical outcomes and might reduce the rate of 12-month major adverse cardiac events when compared with angiography-guided CTO intervention [12, 13]. IVUS images provide information about lesion morphology and contribute to successful procedural results in CTO lesions [12, 14, 15], such as by helping to resolve the ambiguity of the proximal cap [16], facilitating reentry into the true lumen after subintimal crossing [17–19], and helping to find the entry point of the stump [20, 21]. Two catheter types have been used in clinical practice: rotating transducer catheters and electronic array catheters. Unlike the rotating transducer catheters that use a rotating transducer technology, which are predominantly distributed by Boston Scientific, the IVUS type, along with our new device, is an electronic array catheter that is distributed predominantly by the Volcano Corporation (San Diego, CA, USA) and does not need rotation to acquire images. Multiple phased-array elements are oriented circumferentially and receive back-scattered ultrasound signals that are processed into real-time images by using the electronic array design. The IVUS catheter in this device has high push capability and is plug-and-play, whereas most of the rotational transducer IVUS catheters have excellent image quality. The two IVUS types fit through all 5-French or larger guiding catheters.

IVUS supports the guidewire engaged into the occlusion stump in two ways normally. The ideal way is to leave the probe at the side branch or subintimal space, which should provide the best images to guide wire puncture direction. However, there are some limitations of this method in CTO PCI, because the normally used guiding catheter cannot accommodate the microcatheter and IVUS catheter at the same time, so larger guiding catheters are required. The other disadvantage is that the guidewire and microcatheter may be deflected by the IVUS catheter and inhibit contrast injections simultaneously. In this situation, the location of the guidewire has to be reconfirmed; the IVUS has to be reinserted; and the relationship of the puncture target and guidewire has to be modified. The frequent exchange of catheter and guidewire may reduce puncture accuracy and increase the risk of complications. Our new device overcomes these disadvantages. The IVUS catheter used in this device overcomes the limitations of typically used IVUS, such as the inability to offer direct online guidance during the procedure. Instead, this device provides continuous IVUS images. In addition, the microcatheter and the IVUS catheter act as a double-lumen microcatheter to stabilize and support the wiring. If it is hard to find the entry point of a CTO cap, the IVUS catheter in this device can be advanced to the side branch of the occluded lesion via the first wire previously positioned in the side branch, and the device can then be pulled back until the IVUS image shows the entry point of the CTO cap. Next, a CTO-dedicated guidewire can be advanced through the microcatheter lumen to engage the occluded lesion under the guidance of IVUS images. The device and technique show the entry point, which helps the operator choose the right wire, and this significantly increases the successful entry rate of the second wire, which ideally enters centrally. If the guidewire advances into the false lumen of the CTO lesion, the guidewire can be left in the subintimal space, and the IVUS catheter can be advanced into the false lumen along the first guidewire. Next, a CTO-dedicated guidewire can be advanced through the microcatheter lumen to achieve reentry into the true lumen under IVUS guidance. Because the IVUS catheter of this device is introduced into the false lumen, it can occlude the false lumen and act as a marker of the false lumen; moreover, it can smoothen the sharp curve by modifying the anatomy of the coronary artery. In addition, this device also shows the position of the true lumen, the relationship with the first guidewire, the vessel structure, and the pathological features of the CTO lesion, all of which assist the operator in choosing the right wire and help direct the CTO guidewire in the microcatheter into the true lumen.

In conclusion, this new device has the advantages of IVUS and a double-lumen microcatheter, and the CTO-dedicated guidewire can be manipulated precisely and introduced up to the entry point of the true lumen safely and quickly. The device has the potential to be a valuable tool for the recanalization of complex CTO lesions.

We offer the following technique tips:

1. Any side branch from the site of the occlusion can be used if it is large enough to advance an IVUS catheter after gently dilating the side branch if necessary.
2. Choose a 6-French or larger guiding catheter; note the required exchange length of the guidewires if you want to change the wire; and do not use the anchor balloon technique with the 6-French guiding catheter.
3. Advance the IVUS catheter into the side branch deeper and then pull the IVUS catheter back.

## Data Availability

The datasets used and/or analyzed during the current study are available from the corresponding author on reasonable request.

## Abbreviations

CTO: Chronic total occlusion
LAD: Left anterior descending artery
OTW: Over the wire
PCI: Percutaneous coronary intervention
PD: Posterior descending
PL: Posterior lateral
RCA: Right coronary artery
RLS: Real-time intravascular ultrasound double-lumen microcatheter seeking
